# The potential impacts of migratory difficulty, including warmer waters and altered flow conditions, on the reproductive success of salmonid fishes

**DOI:** 10.1016/j.cbpa.2015.11.012

**Published:** 2016-03

**Authors:** Miriam Fenkes, Holly A. Shiels, John L. Fitzpatrick, Robert L. Nudds

**Affiliations:** University of Manchester, Faculty of Life Sciences, Oxford Road, Manchester M13 9PL, United Kingdom

**Keywords:** Salmonids, Migration, Climate change, Urbanisation, Reproduction, Sperm quality, Spawning behaviour, River water temperature, River flow velocity

## Abstract

Climate change and urbanisation of watercourses affect water temperatures and current flow velocities in river systems on a global scale. This represents a particularly critical issue for migratory fish species with complex life histories that use rivers to reproduce. Salmonids are migratory keystone species that provide substantial economical value to ecosystems and human societies. Consequently, a comprehensive understanding of the effects of environmental stressors on their reproductive success is critical in order to ensure their continued abundance during future climatic change. Salmonids are capital breeders, relying entirely on endogenous energy stores to fuel return migration to their natal spawning sites and reproduction upon arrival. Metabolic rates and cost of transport en-route increase with temperature and at extreme temperatures, swimming is increasingly fuelled anaerobically, resulting in an oxygen debt and reduced capacity to recover from exhaustive exercise. Thermally challenged salmonids also produce less viable gametes, which themselves are affected by water temperature after release. Passage through hydrological barriers and temperature changes both affect energy expenditure. As a result, important energetic tradeoffs emerge between extra energy used during migration and that available for other facets of the reproductive cycle, such as reproductive competition and gamete production. However, studies identifying these tradeoffs are extremely sparse. This review focuses on the specific locomotor responses of salmonids to thermal and hydrological challenges, identifying gaps in our knowledge and highlighting the potential implications for key aspects of their reproduction.

## 1 Introduction

Climate change has increased global surface temperatures and is predicted to continue to do so in the future, leading to more frequent hot and fewer cold temperature extremes, intensifying wet and dry seasons, warming the ocean, raising sea levels and affecting ocean circulation (IPCC, 2013). An increased extinction risk is projected especially for terrestrial and freshwater ecosystems, where climate change interacts with additional anthropogenic stressors (IPCC, 2014). Specifically, urbanisation (i.e., building of hydrosystems, removal of riparian vegetation, diversion of river flow for irrigation and hydroelectric power, thermal and chemical pollution) is affecting river systems worldwide, which has strong implications for the health and persistence of associated catchments and biomes (Nilsson et al., 2005). Increases in air temperatures directly affect river water temperatures (Hari et al., 2006, Isaak et al., 2011, van Vliet et al., 2011), altering freshwater ecosystems (IPCC, 2014) and decreasing suitable thermal habitats for aquatic species (Hari et al., 2006, Wenger et al., 2011a, Wenger et al., 2011b), especially cold-adapted fish at the edge of their distribution (e.g. salmonids of the British Isles) (Graham and Harrod, 2009). Recent and future impacts of climate change and urbanisation on freshwater ecosystems force aquatic species to adapt to novel and intensified environmental challenges in order to persist (Isaak et al., 2011, Reed et al., 2011). As a response to increasing temperatures, many species perform poleward or altitudinal shifts in their geographical distribution to seek suitable thermal habitat (IPCC, 2014, Pörtner and Knust, 2007), while others fail to complete large scale migrations (Farrell et al., 2008), which leads to population collapses and local extinctions (Pörtner and Knust, 2007). Adaptation to climatic change and urbanisation represents a particularly critical issue for species with complex life histories, such as migratory fish, where the timing of life cycle transitions is finely tuned to environmental cues and affects subsequent life stages in radically different types of habitat (Crozier et al., 2008). Consequently, migratory fish are at increased risk of extinction compared to non-migratory fish (Cooke et al., 2008b and literature within; Hinch et al., 2005).

As ectothermic poikilotherms, most fish species are strongly affected by water temperature due to its effects on biochemical reactions (Angilletta et al., 2002). Thermal and hydrological conditions affect energetic demands and transport costs in fish (Enders et al., 2003, Enders et al., 2005, Rand et al., 2006, Salinger and Anderson, 2006) and they are crucial factors determining the initiation and success of migrations (Hinch et al., 2005). Low energy reserves at the onset of migration are likely to have significant negative effects on survival and fitness (Crossin et al., 2004), which is exacerbated by stressful migratory conditions such as elevated current flow velocity and water temperature (Rand et al., 2006). Energy depletion during migration inevitably reduces the energetic capital that is retained for subsequent activities, such as reproduction. Little, however, is known about the implications of successful passage through urbanised watercourses and at challenging temperatures on reproductive success upon completion of reproductive migrations (Nadeau et al., 2010).

Salmonids are an ideal model to understand the impacts of climate change and urbanisation on migratory fish physiology, behaviour and reproductive success. They are anadromous or potamodromous migrants moving between distinct habitats as juveniles in search of rich feeding grounds and again as adults when homing upriver to their natal spawning grounds (Groot and Margolis, 1991, Hinch et al., 2005, Jonsson and Jonsson, 2011, Quinn, 2005). Adult salmonids cease feeding upon commencing their spawning migration (Hinch et al., 2005, Kadri et al., 1995, Kadri et al., 1997, Quinn, 2005) and their journey upriver is thus entirely fuelled by endogenous energy reserves (capital breeding) (Crossin et al., 2009). Efficient allocation and expenditure of their limited energy stores is of paramount importance (Crozier et al., 2008, Hinch et al., 2005, Mathes et al., 2010, Young et al., 2006), especially for populations that complete long and arduous migrations (Bernatchez and Dodson, 1987). Unsurprisingly, salmonids have evolved to be highly efficient swimmers (Eliason et al., 2013b). Increased water temperature (e.g. Farrell et al., 2008, Fig. 1), river discharge (e.g. Rand et al., 2006) or a combination of both (e.g. Keefer et al., 2004, Martins et al., 2012) can result in substantial increases in pre-spawning mortality.

However, information about the behavioural and physiological mechanisms determining migratory success is still limited (Caudill et al., 2007, Cooke et al., 2008b). Salmonid migratory mass movements involve high concentrations of individuals following particular pathways (Lucas and Baras, 2001). This concentration of biomass can provide crucial nutrients to otherwise often sparse environments, making them keystone species (Quinn, 2005). Additionally, salmonids are subject to intense commercial and recreational exploitation (Cooke et al., 2008b), creating substantial economic value. Understanding anthropogenic impacts on all aspects of their complex life histories is crucial in order to establish ways to manage their continued abundance and to preserve the ecosystem services they provide.

## 2 Behavioural, physiological and locomotor responses to migratory challenges

**Fig. 2:**
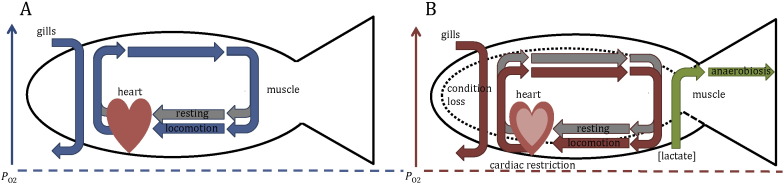
Schematic representing the oxygen cascade of fish during resting and locomotion at water temperatures within thermal tolerance levels (A) and during rest and locomotion at elevated water temperatures (B). A: During rest, O_2_ from the gills is circulated by the heart to sustain basic life functions (standard/basic metabolism, grey arrows). During locomotion (blue arrows), tissue O_2_ demand is increased but this demand can be met by increasing cardiac output and O_2_ uptake at the gills such that metabolism remains within limits of aerobic scope for activity. B: During rest at elevated water temperatures, cardiac and other tissue O_2_ demand is increased and, at extreme temperatures, cannot be met by the cardiorespiratory system, which can lead to recruitment of anaerobic metabolic pathways (grey arrows). This leads to collapse of aerobic scope. During locomotion at elevated water temperatures (red arrows), O_2_ demands are further exacerbated, contributing to O_2_ depletion, cardiac restriction and recruitment of anaerobic metabolic pathways to fuel locomotion. Anaerobic fuelling of locomotion causes lactate build up in muscle tissue (green arrows), anaerobiosis and fatigue, impairing locomotor performance and increasing the metabolic costs of transport. If prolonged, this can lead to energy loss, loss of body condition (dashed line) and/or mortality. Oxygen partial pressure (*P*_O2_) corresponds to the same arbitrary scale in both scenarios (A and B). This scale does not represent exact values, but rather illustrates changes in O_2_ consumption and availability in response to the different environmental stressors and physiological states. For example, PO_2_ levels are lower during rest at elevated temperatures compared to the resting state at cool temperatures, whereas during locomotion at cool temperatures, PO_2_ levels are decreased, similar to the resting state at elevated temperatures.

Adapted from Farrell (2009).

**Fig. 3:**
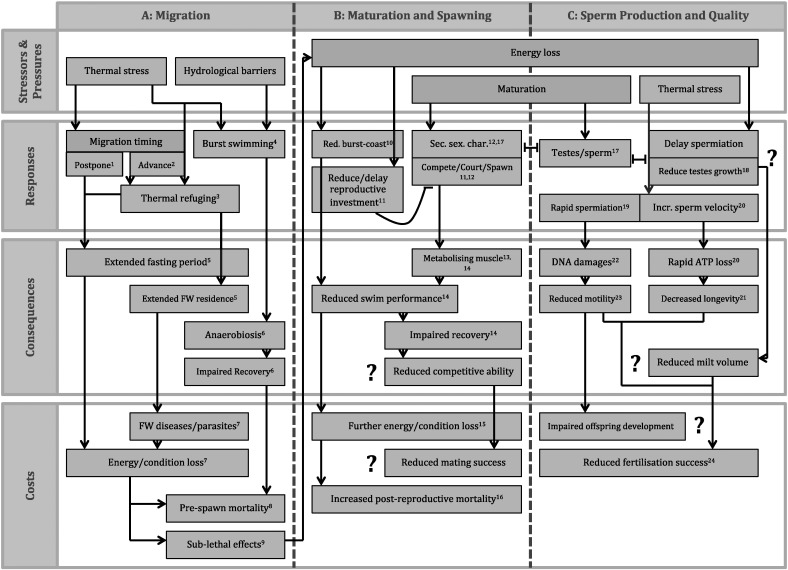
A: The behavioural responses, physiological consequences and energetic/life history costs of thermal stress and hydrological barriers for migrating salmonid fishes. Thermal stress can result in postponement^1^ or advancement^2^ of river entry, delaying migration through thermal refuging^3^ or minimizing exposure by increasing ground speeds through high speed burst swimming^4^. Postponing or advancing river entry as well as thermal refuging extends the reproductive fasting period^5^, increasing energy/condition loss^7^. Advancing river entry and thermal refuging additionally extend freshwater residence^5^, prolonging exposure to freshwater diseases/parasites^7^ and causing further energy/condition loss^7^. Burst swimming^4^ can lead to anaerobiosis^6^, which impairs recovery^6^ and subsequent swim performance, reducing migratory efficiency and causing energy/condition loss^7^. Energy/condition loss, exposure to freshwater diseases/parasites and anaerobiosis can lead to pre-spawn mortality^8^ or may have sub-lethal effects^9^ on subsequent reproduction (detailed in Fig. 3B and C). ^1–9^: references detailed in Table 1. B: The behavioural and physiological responses, potential physiological consequences and energetic, life history and fitness costs of increased energy loss during prolonged fasting and due to stressful migratory conditions for maturing and spawning male salmonids fishes. Salmonids subject to energy loss due to stressful migratory conditions may reduce energy efficient burst-and-coast swimming^10^, reducing subsequent swim performance^14^ and causing further energy/condition loss^15^. Increased migratory stress may reduce/delay reproductive investment^11^, which would impair development of gonads and secondary sexual characters (SSCs)^12^. Reduced investment in reproductive characteristics may be accompanied by a reduction in spawning behaviours and mate acquisition effort. However, maturation exerts physiological “pressure” to develop gonads and SSCs, and to perform spawning behaviours, resulting in a critical tradeoff between energy allocation to fuel migration and subsequent reproduction^12^. Increased energy allocation to gonads/SSCs and spawning behaviours is fuelled through metabolising of muscle tissue^13,14^, which may impair swim performance^14^ in addition to the aforementioned effects of fasting and stressful migrations. Metabolising muscle may further impair recovering abilities^14^, which may lead to a reduction in competitive ability of males during spawning activities. Reduced competitive ability, together with a potential delay or reduction of investment in primary and secondary sexual characteristics may reduce mating success and reproductive fitness. Overall further increased energy/condition loss due to the stressors/pressures of fasting, stressful migrations and maturation may lead to increased post-reproductive mortality^16^ in normally iteroparous species. ^10–16^: references detailed in Table 1. C: The physiological responses, consequences and fitness costs of increased thermal stress as well as energy loss during prolonged fasting and due to stressful migratory conditions during spermiation for sperm quality of salmonid fishes. Testes growth and sperm production may be in an energetic trade off with production of secondary sexual characters^17^. In addition, spermiation may be delayed, due to a potential tradeoff between allocating energy to producing gametes and fuelling migration to reach spawning grounds. In response to prolonged fasting and increased energy loss, testes growth may be reduced^18^ and ultimately, fertilisation success may be compromised^24^. Reduced testes growth and/or delayed spermiation may result in reduced volumes of milt produced, which may add to the reduction in fertilisation success caused by thermal stress. Thermal stress during maturation causes an acceleration of spermiation^19^ as well as higher degrees of DNA damages^22^, which may impact on offspring development and survival. Thermally stressed males produce milt with a reduced percentage of motile sperm^23^. Additionally, thermal stress on sperm after release increases initial sperm velocity^20^, which is at rapid expense of limited ATP stores^20^ and decreases longevity^21^. Reduced motility, longevity of sperm movement and reduced milt volumes are likely to adversely affect fertilisation success in addition to the impacts of male physiological condition^24^. ^17–24^: references detailed in Table 1.

Salmonids have evolved to be highly efficient swimmers and can respond to environmental challenges en route with a variety of avoidance behaviours (e.g., thermal refuging (Mathes et al., 2010) or optimal path selection to avoid high flows (Standen et al., 2004)) and/or energy-minimizing migratory tactics (i.e., kinematic strategies such as burst-and-coast swimming (Videler and Weihs, 1982) or exploiting vortices (Liao et al., 2003, Taguchi and Liao, 2011)). However, thermal and velocity challenges strongly affect salmonid physiology, leading to non-aerobic fuelling of locomotion, anaerobiosis and energy loss when tolerance levels are transgressed (Lee et al., 2003a, Lee et al., 2003b) (Fig. 2). The consequences are often lethal (pre-spawn mortality; e.g., Budy et al., 2002); however, sub-lethal consequences of energy depletion on subsequent reproductive success are highly likely, but seldom considered. The behavioural and physiological responses of salmonids to migratory challenges en route, as well as their physiological consequences and costs, are described in the following and are summarised in Fig. 3A and Table 1.

### 2.1 Behavioural plasticity in migratory tactics

In order to ensure that conditions at the spawning grounds are adequate for reproduction and subsequent offspring development, return migration timing and spawning date are highly conserved traits in salmonids (Crozier et al., 2008, Hodgson and Quinn, 2002, Mathes et al., 2010). However, in response to unusually high river temperatures, some species may advance (Cooke et al., 2008a, Juanes et al., 2004, Quinn and Adams, 1996), while others may postpone (Robards and Quinn, 2002) migration to avoid peak summer temperatures en route (Mathes et al., 2010). Salmonids can also respond to suboptimal water temperatures by delaying migration in cool thermal refuges (Berman and Quinn, 1991, Goniea et al., 2006, High et al., 2006, Hyatt et al., 2003). Thermal refuges include cold water tributaries, lateral groundwater seeps, deep pools and cold alcoves (Caissie, 2006); their location and temporal stability is highly variable (Dugdale et al., 2013) and their preservation will be key in the survivorship of salmonid stocks in the warming climate (Mathes et al., 2010). However, delaying migration through thermal refuging prolongs exposure to freshwater diseases and parasites, which is exacerbated by high water temperatures (Hari et al., 2006) and can contribute to pre-spawning mortality (reviewed by Hinch et al., 2012).

When encountering suboptimal thermal and flow conditions during migration, salmonids exhibit cost minimizing locomotor strategies. Burst-and-coast swimming, where bursts of fast swimming are in cyclic alternation with phases of coasting, in which the body is kept straight and motionless, can be an energetically advantageous strategy that allows fish to gain fast swimming speeds during short bursts while preventing the effects of fatigue by allowing metabolic recovery of muscle fibres during the coast phases (Videler and Weihs, 1982). Migrating fish can conserve energy by identifying and exploiting slow-velocity regions in the water column (Hinch and Rand, 2000, McElroy et al., 2012, Standen et al., 2004) as well as oncoming vortices created by objects in the stream (Liao et al., 2003, Taguchi and Liao, 2011) or by adjusting kinematic movement to maintain swimming efficiency during acute thermal and velocity challenges (Nudds et al., 2014). When confronted with high flow velocities that cannot be avoided, salmonids can adapt their migratory strategy from one of minimizing energy expenditure (seeking low flow regions in the water column) to one of minimizing exposure (rapidly passing through high flow regions by increasing ground speeds) (Standen et al., 2004). This strategy, however, bears other risks: burst swimming in areas of high flow, such as fishways, is facilitated by recruitment of anaerobic muscle fibres and increases subsequent pre-spawn mortality, as seen in sockeye salmon, *Oncorhynchus nerka* (Burnett et al., 2014).

### 2.2 Physiological responses to environmental stressors

At increased temperatures, oxygen demand from life sustaining organs (cardiorespiratory system) increases, which impedes oxygen circulation and limits the metabolic scope for activity (Farrell, 2009), forcing the animal to resort to anaerobic metabolic pathways to survive (Pörtner and Farrell, 2008) (Fig. 2). Swimming at high flow rates also requires anaerobic metabolic pathways to sustain locomotor performance (Burnett et al., 2014). During anaerobic fuelling of locomotion, adenosine triphosphate (ATP) is created via glycolysis in the cytosol, which results in a buildup of lactic acid in the muscle, lowers intrafibre pH (acidosis) and inhibits enzymes of force generation, ultimately causing fatigue (Spurway, 1992). Anaerobic fuelling of locomotion is therefore always time-limited and requires periods of recovery before repeat performance is possible (Farrell et al., 2001, Milligan et al., 2000). When both stressors are combined (elevated temperature and high swim speeds), anaerobiosis leads to cardiorespiratory restriction (Eliason et al., 2013a), limiting locomotor performance (Jain and Farrell, 2003, Lee et al., 2003a, Lee et al., 2003b) and increasing the metabolic costs of transport and energy expenditure (Geist et al., 2003, Lee et al., 2003a, Lee et al., 2003b) (Fig. 2). Because energy reserves are limited in migrating salmonids, the accumulated stress experienced in successive hydrologic challenges and its detrimental effects on body condition and other physiological parameters can result in mortality (Budy et al., 2002). The effect of these hydrologic challenges is exacerbated by concurrently high water temperatures (Martins et al., 2012). Poor body condition at the time of river entry is likely to further intensify the negative effects of dam passage on mortality, as observed in chinook salmon, *Oncorhynchus tshawytscha*, and steelhead trout, *Oncorhynchus mykiss* (Caudill et al., 2007). Moreover, energy-intensive migrations increase physiological stress and induce osmotic imbalances in addition to elevated plasma lactate levels due to anaerobiosis (Nadeau et al., 2010). However, the increased premature recruitment of anaerobic exercise due to variations in river flow and temperature conditions is an important, but understudied factor to consider when studying migratory energetics in salmonids (Lee et al., 2003a, Lee et al., 2003b).

### 2.3 Sub-lethal consequences of energy depletion

The elevated energy depletion resulting from arduous migrations under stressful conditions may have sub-lethal consequences affecting reproductive success upon arrival at the spawning grounds (Fig. 3B, C, Table 1). Salmonids migrate using a finite energy budget, which is used to fuel locomotion and finalisation of gonad and secondary sexual character development during migration as well as spawning activities upon arrival (Kinnison et al., 2003). Adipose tissue reserves are the primary source of energy utilized for upriver migration and gonad production, whereas protein from muscular tissue serves to fuel development of secondary sexual characters as well as metabolism during spawning activities (Hendry and Berg, 1999). For example, white muscle protein and enzyme activities decrease drastically in migrating sockeye salmon, suggesting that white muscle is metabolized (Mommsen et al., 1980). Therefore, in addition to driving locomotion, muscle acts as fuel and contributes to overall reproductive investment. Reduced muscle mass and body condition can negatively affect swimming performance, as seen in cod, *Gadus morhua*, who perform poorer in terms of endurance swimming (prolonged swimming at speeds above *U*_crit_) and implement energy efficient burst-and-coast swimming less frequently when starved (Martinez et al., 2003). In the broadcast spawning scallops *Euvola ziczac* and *Chlamys islandica*, increased reproductive investment is correlated with decreased muscle mitochondrial oxidative capacities and increased time to recover from exhaustive exercise (Boadas et al., 1997, Brokordt et al., 2000). To avoid interference with swimming ability during migratory movement, development of secondary sexual characters at the expense of muscle protein is postponed to later stages of migration in salmonids. However, acutely increased energy requirements during migration, for example, through intensified thermal and hydrological challenges, may nevertheless be supplied at the expense of energy available for gamete production, secondary sexual character development and reproductive activity (Hendry and Berg, 1999) and vice versa (see Fig. 3B, C and Table 1).

## 3 Locomotor performance and reproductive investment: a trade off

For both female and male salmonids, somatic mass and body size are positively correlated with dominance during spawning activities (Fleming, 1998, Fleming et al., 1996, Fleming et al., 1997). However, larger males may incur higher costs of transport and subsequent energy loss when subjected to thermal challenges and this may be exacerbated during passage through urbanised rivers. Energetic tradeoffs exist between fuelling migration and investment in secondary sexual characters (Kinnison et al., 2003, Fig. 4). Similar tradeoffs may impair male mate acquisition effort upon arrival, which may ultimately impact on their reproductive success. The following section describes these energetic trade-offs in detail, highlighting existing knowledge gaps. Fig. 3 and Table 1 detail the potential responses to and sub-lethal consequences and costs of energy depletion en route for male salmonid reproductive effort and success.

### 3.1 The determinants of salmonid reproductive success

Salmonids are external fertilisers that reproduce by releasing gametes into the water during paired matings (Esteve, 2005). Female salmonids maximise their reproductive fitness by competing for access to suitable nest sites (Esteve, 2005) and female reproductive success is highly correlated with somatic mass and gamete size: large females are able to secure better quality nesting sites (Hendry et al., 2001) and can dig deeper redds (Blanchfield and Ridgway, 2005, Crisp and Carling, 1989, Fleming, 1998, Fleming et al., 1997), which are better protected against destructive gravel shifts, river desiccation, freezing and nest superimposition by other females (Fleming, 1998). Larger females also produce larger eggs (Fleming, 1996, Hendry et al., 2001, Quinn et al., 1995) and large eggs yield large fry and juveniles, which exhibit higher growth and survival rates due to their superior competitive abilities and reduced predation risk compared to smaller juveniles (Einum and Fleming, 1999, Einum and Fleming, 2000, Hendry et al., 2001, Hutchings, 1991). Males maximise their reproductive fitness quantitatively, by competing for, and mating with, multiple females (Esteve, 2005). Territorial males fight ferociously among themselves for access to reproductively active females, combining actual fighting with intimidating threat displays (Esteve, 2005) and ultimately forming a dominance hierarchy (Chapman, 1966, Grobler and Wood, 2013). In order to maximise paternity further, males attempt to secure and maintain the closest possible vicinity to females and their eggs (Fitzpatrick and Liley, 2008, Maekawa and Onozato, 1986, Mjolnerod et al., 1998, Tuset et al., 2008) and/or release sperm of superior quality in terms of volume, velocity and viability (Simmons and Fitzpatrick, 2012). Body size and morphology are strong predictors of male dominance acquisition: the majority of salmonid matings involve single large, dominant males (Fleming et al., 1996, Fleming et al., 1997) achieving superior rates of paternity [chinook salmon (Schroder et al., 2011) and masu salmon, *Oncorhynchus masou* (Watanabe and Maekawa, 2010)]. Fork length and the size of their dorsal hump (a secondary sexual characteristic) positively affect social status in sockeye salmon (Quinn and Foote, 1994, Quinn et al., 2001), and brown trout, *Salmo trutta* (Jacob et al., 2007) and access to reproductively active females is positively correlated with dominance in brook trout, *Salvelinus fontinalis* (Blanchfield et al., 2003) and body size in pink salmon, *Oncorhynchus gorbuscha* (Dickerson et al., 2002). Additionally, females favour large males, either delaying oviposition [chinook salmon (Berejikian et al., 2000), brook trout (Blanchfield and Ridgway, 1999), Atlantic salmon, *Salmo salar* (Gaudemar et al., 2000) and sockeye salmon (Foote, 1988, Foote, 1989, Foote and Larkin, 1988)] or actively attacking undesirable, small males [coho salmon, *Oncorhynchus kisutch* (Berejikian et al., 1997), Atlantic salmon (Järvi, 1990) and sea trout, *S. trutta* (Petersson and Jarvi, 1997)].

### 3.2 Energetic consequences of dominance in male salmonids

The behavioural suite of territorial spawning males is highly energetically demanding: higher activity in terms of aggressive interactions of dominant males causes higher rates of physiological condition loss compared to less active, subordinate males (Healey et al., 2003, Hendry and Beall, 2004). Males arrive earlier (protandry; Morbey, 2000, Morbey, 2003) and stay active for longer at the spawning grounds than females (Fleming et al., 1996, Fleming et al., 1997), continuously competing for mates in the meantime (endurance rivalry). Larger males may perform better (i.e. longer) in endurance rivalry due to their higher maximum energy storage capacity compared to smaller males (Esteve, 2005). However, although larger bodied animals may incur lower transport costs compared to smaller conspecifics (Rodnick et al., 2004, Schmidt-Nielsen, 1972), larger individuals of Atlantic salmon were shown to suffer greater total energy loss due to migration and spawning than smaller conspecifics (Jonsson et al., 1997). This may be due to a positive relationship between energy allocation to reproductive traits and body size, which has been described for Arctic charr, *Salvelinus alpinus* (Dutil, 1986) at the expense of somatic energy. Moreover, studies on chinook salmon (Clark et al., 2008), coho salmon (Clark et al., 2012) and redband trout, *O. mykiss* (Rodnick et al., 2004) have demonstrated a reduced tolerance to an acute thermal challenge in larger compared to smaller individuals. Therefore, when exposed to elevated water temperatures, the high rates of activity exhibited by large dominant males may ultimately impair their ability to recover from repeated competitions and compromise their fighting ability for the duration of the spawning season (Fig. 3B). Increased energy use has been shown to reduce post-reproductive survival (Berg et al., 1998, Jonsson et al., 1997) and to increase the tendency for larger fish to spawn biennially rather than annually (Jonsson et al., 1991). Large body size presents males with an advantage for mate acquisition, but it may impose disproportionately high strain on their energy balance, with possible detrimental consequences for future reproductive success in iteroparous species that should be capable of reproducing multiple times. In order to meet energetic demands during long-distance migrations, salmonids tend to prolong pre-spawning growth phases: in Atlantic salmon, mean body size and age at maturity increase with the length and mean annual discharge of the rivers they navigate en route to their spawning grounds (Jonsson et al., 1991). Body size similarly increases with migratory distance for returning anadromous brown trout in Norway (Jonsson and Jonsson, 2006), as does age at first spawning, which is correlated with body size in Atlantic salmon in North America (Schaffer and Elson, 1975). These results suggest that tradeoffs exist between fuelling arduous migrations and reproduction, and that body morphology may play a crucial role in determining the direction and extent of investment in one over the other. In an era of climate change and urbanisation, increased body size may ultimately impair migratory and reproductive success, potentially adding to the existing pressures of size-discriminative predation (Quinn and Kinnison, 1999) and fishing (Cooke et al., 2009, Kendall and Quinn, 2013) on larger individuals. To date, however, spawning behaviour and competitive ability of male salmonids as affected by migratory stress through suboptimal environmental conditions have not been studied.

### 3.3 Effects of arduous migrations on reproductive investment

A number of studies have found tradeoffs between migratory rigour and other aspects of reproductive investment in salmonids. Female chinook salmon subjected to an experimental increase in migratory distance and elevation incurred higher somatic energy costs (~ 17% decrease in metabolizable body mass) and showed a decrease in their gonadal investment (~ 14% reduced ovarian mass) in the form of reduced egg size (Kinnison et al., 2001). Palstra et al. (2010) compared sexual maturation progress between experimentally exercised female rainbow trout, *O. mykiss*, and females kept in still water. They showed that exercise inhibits vitellogenin uptake and results in reduced numbers of late vitellogenic oocytes, leading the authors (Palstra et al., 2010) to conclude that during long reproductive migrations, energy is reallocated to fuel exercise, suppressing ovarian developmental progress. One possible explanation for a reduction in gonadal investment with increased migratory costs surrounds altered hydrodynamics and energetics of females with large ovaries, which may reduce swimming efficiency, energetic capital and, ultimately, survival (Kinnison et al., 2001). Additionally, in a study on captive female masu salmon, Mingist et al. (2007) demonstrated that increased cortisol levels, which can be a result of difficult, stressful migrations (Nadeau et al., 2010), decrease the number of eggs developed to eyed egg stage.

In males, experimentally increased migration distance and elevation negatively affect the expression of secondary sexual characters (dorsal hump size and upper jaw length), reduce somatic energy reserves upon arrival at the spawning grounds (chinook salmon; Kinnison et al., 2003) and male gonadal mass declines with migration distance (brown trout; Jonsson and Jonsson, 2006). Similar to females with larger ovaries, a greater deviation from a fusiform shape due to the expression of secondary sexual characters may increase costs of transport and reduce migratory efficiency in male salmonids (Kinnison et al., 2003). However, the specific effects of this energy deficit on spawning behaviour and competitive ability as well as sperm quality are currently unknown.

## 4 Does migratory effort affect male gamete quality?

Even though dominance in male salmonids is positively correlated with somatic mass, body size and/or the expression of secondary sexual characters, dominant males do not necessarily monopolise fertilisation of their female partner's eggs. The high degree of sperm competition governing males in disfavoured mating roles can lead to an increased investment in sperm quality and ejaculate volume, enabling subdominant males to compensate for their inferior mate acquisition capabilities (Haugland et al., 2009, Rudolfsen et al., 2006). In addition, potentially higher rates of energy loss for large, dominant males compared to subdominant males may lead to reductions in dominant male fertilisation success. The potential links between migratory energetics, body condition and sperm quality, with implications for salmonid reproductive fitness, are discussed in the following and are summarised in Fig. 3C and Table 1.

### 4.1 Sperm competition and the factors affecting fertilisation success

Individual fertilisation probability during competitive spawning in external fertilisers is influenced by four main mating/sperm characteristics (Taborsky, 1998): proximity to eggs during oviposition [brook trout (Blanchfield et al., 2003) and Atlantic salmon (Mjolnerod et al., 1998)], timing and coordination of sperm release with egg release [rainbow trout (Fitzpatrick and Liley, 2008) and sockeye salmon (Hoysak and Liley, 2001), but see Hoysak et al. (2004)], ejaculate volume [bluegill sunfish, *Lepomis macrochirus* (Neff et al., 2003) but see Gage et al. (2004)] and sperm motility characteristics such as swimming speed (Atlantic salmon; Gage et al., 2004) as well as tradeoffs between these traits (e.g. energetic tradeoff between behavioural effort and gonadal investment; Taborsky, 1998). Males who secure the dominant position (favoured mating role) among a group of males competing to fertilise a given set of eggs only experience sperm competition when other males are present, whereas males who cannot secure access to a female have to mate as satellites or sneakers (disfavoured mating role) and experience high levels of sperm competition at all times (Fitzpatrick et al., 2007). Given that large males are usually more likely to secure favoured mating roles, the first three of Taborsky's (1998) determinants of fertilisation success (proximity to eggs, sperm release timing and ejaculate volume) are likely to scale positively with male body size. Yet, smaller males in disfavoured roles can compensate for their inferior mating position with superior sperm motility traits: sperm from subdominant males is faster in cichlids, *Telmatochromis vittatus* (Fitzpatrick et al., 2007) and Arctic charr (Haugland et al., 2009, Rudolfsen et al., 2006). Milt yields of subdominant Arctic charr were larger, suggesting rapid adjustment to mating in disfavoured roles and greater investment in ejaculates compared to males in favoured roles [(Rudolfsen et al., 2006), but see Kortet et al. (2004)]. In coho salmon, sperm quality parameters among large territorial males are negatively correlated with the expression of secondary sexual characters: males with a more intense spawning colouration have lower sperm velocities than males with less intense colouration, suggesting an energetic trade-off between investment in sexual colouration and sperm quality (Pitcher et al., 2009). While large body size provides advantages in male breeding competition, it does therefore not necessarily translate into improved sperm quality. The migratory experience of males and energetic constraints and trade-offs in response to this experience are additional important factors that need to be considered but have rarely been described.

### 4.2 Effects of temperature on sperm quality

Gamete development and spawning in salmonids are endocrinologically regulated and controlled by prevailing photoperiod (Migaud et al., 2010) and temperature regimes (Pankhurst and King, 2010). For instance, within the natural thermal window of the species, increased water temperature accelerates vitellogenesis and oocyte development [Atlantic salmon (Olin and Vonderdecken, 1989) and rainbow trout (Mackay and Lazier, 1993)], whereas transgression of the thermal optimum inhibits these processes [Atlantic salmon (Taranger and Hansen, 1993) and rainbow trout (Pankhurst et al., 1996)]. Similarly, levels of gonadal steroids (testosterone and 11-ketotestosterone) are elevated in male rainbow trout held at 12 °C versus 6 °C (Manning and Kime, 1985), indicating acceleration of spermatogenesis with increased temperature.

Teleost fish sperm differs from that of other animals in that teleost sperm cells are immotile upon ejaculation, motility is induced in contact with water and lasts for less than two minutes (Kime et al., 2001). Therefore, conditions of the activation medium (e.g. water temperature and pH) can affect sperm movement (Alavi and Cosson, 2005). Water temperature affects motility, velocity, longevity and fertilisation capacity of salmonid sperm directly (Alavi and Cosson, 2005, Billard and Cosson, 1992, Lahnsteiner, 2012) as well as indirectly through the thermal experience of males (Alavi and Cosson, 2005, Lahnsteiner and Kletzl, 2012, Lahnsteiner and Leitner, 2013, Williot et al., 2000). For example, the percentage of motile sperm decreased with increasing holding tank temperature in Siberian sturgeon, *Acipenser baeri* (Williot et al., 2000) and the length of spermatozoa motility is decreased at increasing temperature of the activation medium in Atlantic salmon, but not in brown trout (Vladic and Jarvi, 1997), emphasising the highly complex nature of temperature influences on sperm quality parameters. Sperm flagellum beat frequency increases, but motility duration concurrently decreases with increasing temperature of the activation medium in rainbow trout, resulting in a tradeoff between sperm velocity and longevity due to rapid depletion of ATP stores (Billard and Cosson, 1992). Furthermore, increasing temperatures can reduce the percentage of motile spermatozoa in a sample of milt (Cosson et al., 1985, Cosson et al., 1995, Lahnsteiner, 2012) and decrease sperm motility (Cosson et al., 1985, Cosson et al., 1995, Lahnsteiner, 2012, Mehlis and Bakker, 2014). Males experiencing higher temperatures in their holding tanks show reduced maturation rates and gamete quality (Lahnsteiner and Kletzl, 2012, Perchec et al., 1995, Vladic and Jarvi, 1997), as well as a shift and shortening of peak spawning activity and an increase in DNA damaged spermatozoa (Lahnsteiner and Leitner, 2013). Overall, however, data on the effects of thermal experience on sperm quality is lacking.

### 4.3 Gamete quality in response to migratory experience

The pre-spawning experience en route to their spawning grounds in terms of length of migration as well as the frequency and severity of hydrological challenges strongly affect salmonid physiology, depleting their limited energy stores. Similar to the marked effects of arduous migrations on maturation, investment in reproductive characteristics and potentially reproductive behaviour, physical exhaustion during gamete production prior to spawning may impair gamete quality in salmonids (Fig. 3C). In lake whitefish, *Coregonus clupeaformis*, body condition prior to spawning affects ejaculate investment: larger, healthier males produce larger testes; and sperm energetic status in the form of ATP content is increased in males with larger testes (Burness et al., 2008). This suggests that in turn, poor body condition potentially reduces investment in testes mass and sperm quality, which can result in poorer fertilisation success (Rakitin et al., 1999) and may impair offspring survival (Lahnsteiner and Leitner, 2013). Sperm motility can be reduced with the advancement of the reproductive season in rainbow trout (Munkittrick and Moccia, 1987), as well as a number of non-salmonid species (Alavi et al., 2010, Babiak et al., 2006, Golpour et al., 2013, Rideout et al., 2004, Rouxel et al., 2008, Suquet et al., 1998), suggesting that prolonged breeding activity, such as through delayed migration in response to unfavourable conditions, may negatively impact on gamete quality. However, whether changes in gametogenesis and gamete quality over the course of the spawning period are connected to energetic constraints in relation to migration length and difficulty is yet to be considered in male migratory fish such as salmonids. Future research in this area may yield vital information towards the sustainable management of salmonid stocks.

## 5 Conclusions and future directions

Body size and body condition strongly affect salmonid physiology during their catabolically fuelled spawning migration and subsequent reproduction. Larger fish can achieve higher swimming speeds and metabolic efficiency. Large males enjoy advantages during breeding competition and can gain higher fertilisation rates due to their precedence over access to females as well as higher ejaculate volumes available from larger testes. Body condition positively influences swimming as well as reproductive performance due to the higher levels of available stored energy, and body condition positively affects sperm quality. However, transgressions of the optimal temperature range have profound impacts on fish physiology: metabolic rates and cost of transport increase with temperature and swimming has to be increasingly fuelled anaerobically, resulting in reduced capacity to recover from exhaustive exercise. Salmonid spawning behaviour is energetically demanding and is performed by fish with dwindling energy reserves. Increased thermal sensitivity of larger males may affect their performance in intra-sexual competition and may shift selection pressures to ultimately disfavour the top end of the size distribution. However, the effects of altered water temperatures on male competitive ability have not been studied. Thermally challenged males produce less viable sperm and spermatozoa are additionally affected by water temperature after release. These negative effects may be exacerbated by higher loss of energy stores during increasingly challenging migrations through urbanised rivers at higher temperatures. The effects of migratory experience on male competitive ability and gamete quality, however, remain unknown. Salmonid reproduction is highly complex and therefore prone to failure when conditions are suboptimal. We know with reasonable accuracy how environmental conditions affect migration success and survival. Salmonid reproductive effort, however, does not end with their successful arrival at the spawning grounds. We suggest that, in order to enhance our understanding of the effects of anthropogenic disturbances on salmonid populations, future research has to encompass all aspects of their reproduction, incorporating interdisciplinary studies of migratory and reproductive physiology as well as behaviour. Only by considering all aspects of their reproduction can effective ways be found to manage and secure their continued abundance in the face of current and future climatic change and urbanisation.

## Figures and Tables

**Fig. 1:**
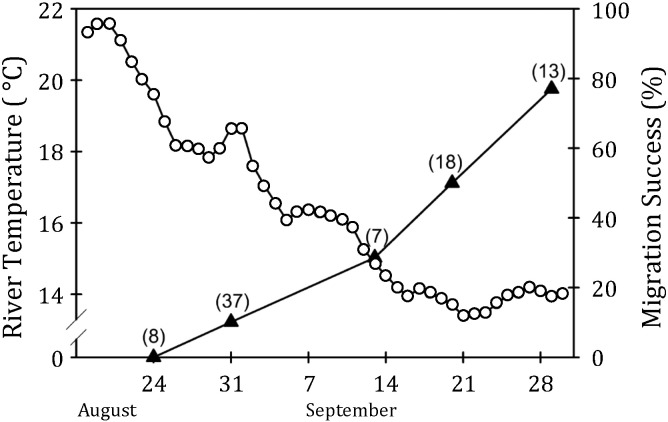
Changes in river temperature and migration success of Weaver Creek sockeye salmon, *Oncorhynchus nerka*, migrating through Harrison River, CA, in 2004. Lines and triangles denote survival rates (%); continuous line and circles denote water temperature (°C). Individual salmon were intercepted and fitted with radio transmitters; the number of tagged and released fish is shown in parentheses for each of five tagging dates (indicated by triangles). Mean (± 95% confidence interval) entry dates are based on previous telemetry results showing migration rates of 25–36 km/d for tagged Weaver Creek sockeye salmon. Due to extremely high river temperatures in 2004, low overall migration success (30%) was observed. However, individual migration success increased with decreasing temperature and was highest (78%) for salmon sampled at the last tagging date; these fish delayed river entry by holding in the cooler estuary for several weeks and migrated when temperatures in the river had decreased. The results indicate the detrimental effects of early river entry at near critical water temperature.

Redrawn with permission from Farrell et al. (2008).

**Fig. 4:**
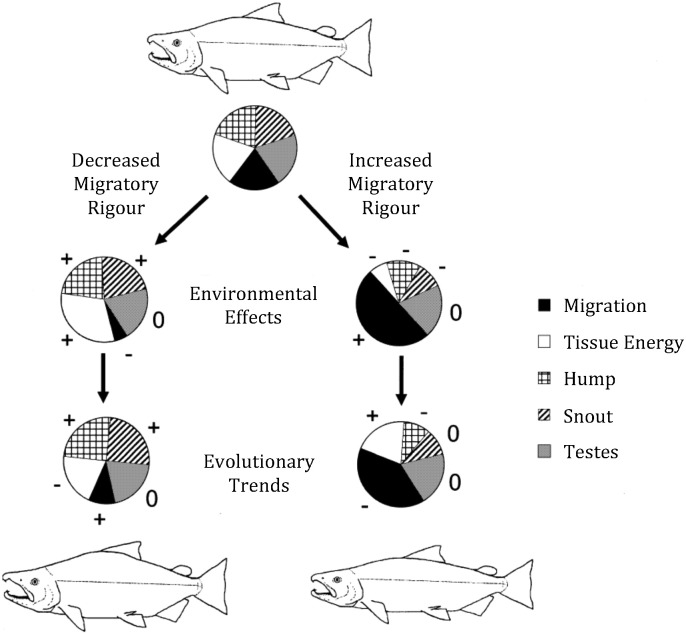
Schematic illustrating predicted environmental and evolutionary effects of altered migratory rigour on reproductive investment and evolutionary trends in secondary sexual character development of male salmon. Wedge sizes are not exact but signify changes in bin sizes in response to environmental effects and evolutionary trends under the given altered migratory costs: +, increase in bin size; 0, no change; −, decrease in bin size. Predictions were empirically supported by a large-scale, long-term relocation experiment conducted with tagged male chinook salmon, *Oncorhynchus tshawytscha*, released and recovered at spawning sites on South Island, NZ. Hump and snout size were reduced and survival rates were lower in males after completing more arduous migrations compared to males from the same populations migrating less far and with less elevation, showing a distinct phenotypic cost of migration in reproductive traits. An effect of altered migratory rigour on gonad development was not detected in this experiment. A strong decrease in tissue energy was attributed to deviation from a fusiform shape in mature males expressing secondary sexual characters, leading to an evolutionary trend of reduced character expression for populations with longer migrations.

Redrawn with permission from Kinnison et al. (2003).

**Table 1:** Key literature concerning the known and potential effects of thermal stress, hydrological barriers and energetic status on migrating, maturing male salmonid reproduction. Numbers correspond to superscripts in Fig. 3. Questions refer to current knowledge gaps outlined in relevant sections of the text. Refer to the text and Fig. 3 for further detail.

A: Migration						
Stressors & pressures	Thermal stress			Hydrological barriers		
	Species	Reference		Species	Reference
Responses	1) Postpone migration timing	Sockeye salmon	Cooke et al. (2008a); Quinn and Adams (1996)	4) Burst swimming in areas of high flow	sockeye salmon	Burnett et al. (2014); Standen et al. (2004)
Atlantic salmon	Juanes et al. (2004)			
2) Advance migration timing	Steelhead trout	Robards and Quinn (2002)
3) Thermal refuging	Chinook salmon	Berman and Quinn (1991); Goniea et al. (2006)
Steelhead trout	High et al. (2006)
Sockeye salmon	Hyatt et al. (2003)
Consequences	5) Extended fasting period & fresh water residence	Sockeye salmon	Hinch et al. (2012)	6) Anaerobiosis & impaired recovery after swimming at high flow (and elevated temperature*)	Sockeye salmon	Burnett et al. (2014); Lee et al. (2003a⁎); Lee et al. (2003b⁎)
			Coho salmon	Lee et al. (2003a⁎); Lee et al. (2003b⁎)
Rainbow trout	Jain and Farrell (2003⁎)
Chinook salmon	Geist et al. (2003⁎)
Costs	7) Exposure to FW diseases & parasites; energy & condition loss	Sockeye salmon	Hinch et al. (2012)			
Brown trout	Hari et al. (2006)
8) Pre-spawn mortality caused by arduous migrations (thermal & hydrosystem experience)	Sockeye salmon	Burnett et al. (2014); Hinch et al. (2012); Martins et al. (2012)			
Chinook salmon & steelhead trout	Caudill et al. (2007)			
*Oncorhynchus* spp.	Budy et al. (2002)			
9) Sub-lethal effects of stress through arduous migrations & energy loss	*Oncorhynchus* spp.	Budy et al. (2002)			
Sockeye salmon	Nadeau et al. (2010)			

B: Maturation & spawning						

Stressors & pressures	Energy loss			Maturation		

	Species	Reference		Species	Reference

Responses	10) Starvation reduces energy-minimizing burst-and-coasting	Cod	Martinez et al. (2003)	12) Energetic trade-off to fuel migration, SSC development and reproduction	Chinook salmon	Kinnison et al. (2003)
11) Energy-intensive migration at a cost for reproductive investment	Sockeye salmon	Hendry and Berg (1999)	Brown trout	Jonsson and Jonsson (2006)
Consequences	13) Metabolising muscle to fuel migration	Sockeye salmon	Mommsen et al. (1980)	14) Reproductive investment reduces locomotor performance & recovery	Scallops	Boadas et al. (1997); Brokordt et al. (2000)
Does energy loss during migration affect male competitive ability and courtship effort?					
Costs	16) Increased energy use increases post-reproductive mortality	Atlantic salmon	Jonsson et al. (1997)	15) High reproductive investment (dominance/aggression) causes energy loss	Atlantic salmon	Hendry and Beall (2004)
Brown trout	Berg et al. (1998)	Sockeye salmon	Healey et al. (2003)
Does reduced reproductive investment due to migratory experience and energy loss impair male mating success?					


C: Sperm production and quality						
Stressors & pressures	Energy loss			Thermal stress		
	Species	Reference		Species	Reference
Responses	17) Energetic trade-off between ejaculate quality and SSCs	Coho salmon	Pitcher et al. (2009)	19) Accelerated spermiation at increased holding temperature	Rainbow trout	Manning and Kime (1985)
18) Poor body condition reduces testes growth	Lake whitefish	Burness et al. (2008)	20) Increased sperm velocity at high activation temperature leads to rapid ATP loss	Rainbow trout	Billard and Cosson (1992)
Does energy loss cause delayed spermiation and reduced investment in testes?				
Consequences				21) Decreased sperm longevity with increased water temperature	Atlantic salmon	Vladic and Jarvi (1997)
Does energy loss and reduced investment in testes reduce ejaculate quality and milt volume?			Rainbow trout	Billard and Cosson (1992)
			22) DNA damaged spermatozoa in warm acclimated males	Brown trout	Lahnsteiner and Leitner (2013)
23) Reduced motility with increasing temperature	Rainbow trout	Cosson et al. (1985); Cosson et al. (1995)
Brown trout	Lahnsteiner (2012)
Costs	24) Male body condition affects sperm fertilisation success	Atlantic cod	Rakitin et al. (1999)			
Do the effects of thermal stress and energy loss on male reproductive investment impair offspring development and survival?					
